# Correlation between imaging and histology in benign solitary retroperitoneal nerve sheath tumors: a pictorial review

**DOI:** 10.1186/s13244-024-01709-5

**Published:** 2024-05-31

**Authors:** Luisa Carone, Gaia Messana, Alessandro Vanoli, Luigi Pugliese, Anna Gallotti, Lorenzo Preda

**Affiliations:** 1 Radiology 1 Unit, IRCCS San Matteo Hospital Foundation, Viale Camillo Golgi, 19, 27100 Pavia, Italy; 2 Diagnostic Imaging and Radiotherapy Unit, Department of Clinical, Surgical, Diagnostic, and Pediatric Sciences, University of Pavia, Viale Brambilla, 74, 27100 Pavia, Italy; 3 Department of Molecular Medicine, University of Pavia, Viale Camillo Golgi, 19, 27100 Pavia, Italy; 4 Unit of Anatomic Pathology, IRCCS San Matteo Hospital Foundation, Viale Camillo Golgi, 19, 27100 Pavia, Italy; 5 General Surgery 2 Unit, IRCCS San Matteo Hospital Foundation, Viale Camillo Golgi, 19, 27100 Pavia, Italy

**Keywords:** Retroperitoneal neoplasms, Schwannoma, Neurofibroma, Multidetector computed tomography, Magnetic resonance imaging

## Abstract

**Background:**

Benign nerve sheath tumors presenting as solitary retroperitoneal masses (RBNSTs) pose a complex diagnostic challenge for multidisciplinary teams regarding differential diagnosis, staging, and treatment planning. This article reviews the role played by different imaging techniques in assessing RBNSTs and elucidates their typical pathological features with a particular emphasis on the correlation between imaging and histological findings. Furthermore, some examples of retroperitoneal tumors that merit consideration in the process of differential diagnosis based on cross-sectional investigations (CSIs) are reported. The correlation between tissue architecture and appearance on imaging can help increase the accuracy of differential diagnosis with other retroperitoneal neoplasms at CSIs.

**Critical relevance statement:**

This educational review critically examines the correlation between imaging and histological features in solitary retroperitoneal benign nerve sheath tumors, offering valuable insights for improving the accuracy of differential diagnosis in clinical radiology.

**Key Points:**

- RBNSTs are challenging to diagnose because they lack specific radiological features.
- Differential diagnosis of RBNSTs from other retroperitoneal neoplasms on imaging is complex.
- Surgical removal of RBNSTs is recommended for an accurate diagnosis.

**Graphical Abstract:**

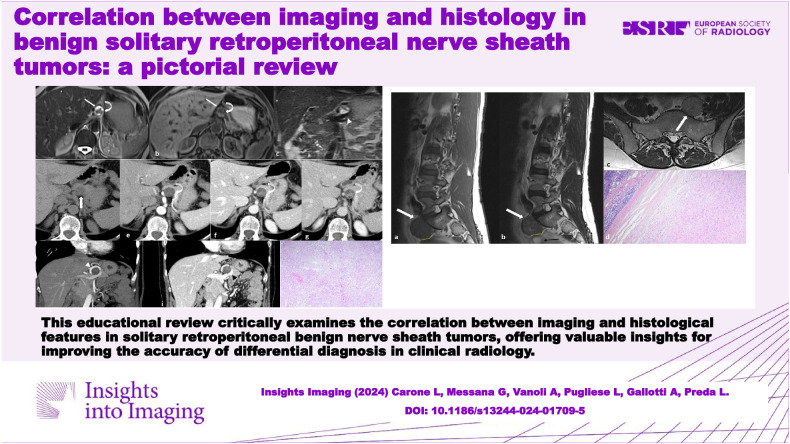

## Introduction

Retroperitoneal benign nerve sheath tumors (RBNSTs) are rare neoplasms that account for about 5% of all retroperitoneal tumors and up to 12% of all benign soft tissue tumors [1, 2]. In the retroperitoneum, BNSTs are mainly represented by Schwannomas and, less frequently, neurofibromas [1].

Schwannomas are more prevalent between the second and fifth decades of life and typically present as sporadic masses, although can occasionally be associated with genetic syndromes such as type 2 neurofibromatosis.

Neurofibromas are solitary in up to 90% of cases and typically not associated with type 1 neurofibromatosis (NF1), instead, multiple neurofibromas or plexiform neurofibromas are nearly diagnostic of NF1. The age of onset in patients with solitary neurofibromas is 20 to 30 years old, but neurofibromas in patients with NF1 often present at an earlier age [1]. Neurofibromas, differently from Schwannomas, are often unencapsulated [3].

Malignant degeneration is rare in both lesions.

The majority of RBSNTs typically present as solitary masses in various locations of the retroperitoneal space incidentally discovered at routine cross-sectional imaging (CSI) performed for other reasons or for symptoms not directly related to their presence. Despite their indolent behavior and negligible risk of metastatic spread, correct identification of these lesions is essential to rule out several alternative diagnoses, including malignancies (e.g., sarcoma, metastatic adenopathy, cystic lymphangioma, paraganglioma, gastrointestinal stromal tumors, etc.).

Although they have typical pathologic and molecular features, they lack specific radiological features that facilitate their distinction from sarcomas and other retroperitoneal cancers. The primary objective of the present review is to highlight the correlation between the histological features and the corresponding radiological characteristics of RBNSTs. This endeavor aims to potentially increase the accuracy of differential diagnosis when encountering other retroperitoneal neoplasms during cross-sectional imaging.

## Pathological features

Macroscopically, RBNSTs usually show solid structure, with possible sparse focal cystic areas. Schawannomas are typically encapsulated and tend to grow eccentrically in relation to peripheral nerve fibers, while neurofibromas often lack a well-defined capsule [4, 5].

Microscopically, Schwannomas consist of admixed hypercellular “Antoni A” areas, characterized by benign neoplastic Schwann cells, and loose hypocellular “Antoni B” zones. “Antoni A” areas commonly exhibit Verocay bodies, which are defined as nuclear palisading around cellular fibrillary processes [4]. On immunohistochemistry, diffuse expression of S100 and SOX10 by the tumoral cells is typical. In addition, neoplastic cells may express calretinin, whereas CD34 is negative or only focally positive [6].

Neurofibromas are composed of a mix of Schwann cells, perineurial-like cells, and fibroblasts, interspersed with nerve fibers, wire-like strands of “shredded carrot” collagen, and myxoid matrix; scattered mast cells are commonly seen [4]. In both tumor types, neoplastic cells lack substantial mitotic activity [7].

## Radiological features on ultrasound

Ultrasound (US) plays a marginal role in the evaluation of RBNSTs, especially in cases of smaller lesions or robust individuals, because of its limited ability to explore the retroperitoneum. Nonetheless, it remains a useful tool in the initial assessment and monitoring of these tumors.

US can provide information about their size, location, morphological features, the presence or absence of cystic components and calcifications, as well as the degree of vascularity based on color-Doppler imaging. RBNSTs typically appear as well-defined, often encapsulated, round masses with posterior acoustic enhancement, which can vary in echogenicity from hypoechoic to heterogeneously echogenic, depending on their composition. They may exhibit cystic areas and focal calcifications, the latter characterized by posterior shadowing [8–10]. In addition, some authors have described their possible ultrasonography target appearance, with a hyperechoic central area and hypoechoic periphery [11]. On color-Doppler evaluation, they frequently show increased vascularity, particularly in the case of Schwannomas [10]. Moreover, US can be helpful in assessing the involvement of surrounding organs and vascular structures; however, it may not accurately demonstrate relationships with other organs as do cross-sectional imaging modalities.

US is a cost-effective and widely accessible imaging modality that does not use ionizing radiation. Nonetheless, for a comprehensive evaluation and characterization of these tumors, additional imaging techniques are necessary.

## Radiologic features on cross-sectional investigations and correlation with pathology

Computed tomography (CT) and magnetic resonance imaging (MRI) are the primary imaging modalities for the diagnosis and follow-up of RBNSTs.

RBNSTs are usually located in the paravertebral region, close to the inferior vena cava and aorta, and less commonly, adjacent to the kidney, pre-sacral space, around the porta hepatis, and/or abdominal wall [12]. Within the retroperitoneal region, the presence of a mass characterized by a smooth expansion, that originates in proximity to the spine, in the context of the psoas muscle, or that affects the neural foramina devoid of evident bony destruction, indicates a potential benign neural origin; however, these findings are not specific.

When a RBNST is localized in proximity to the duodenum or pancreas, identifying the precise organ of origin on CSI can be challenging. Indicative signs such as the “beak sign”, wherein a mass distorts the edge of an adjacent organ into a distinctive “beak” shape, the “phantom organ sign”, which occurs when a mass emerges from a smaller organ causing the latter to appear indistinct, and the “embedded organ sign”, where segments of an organ seem embedded within the tumor, may be indicative of a duodenal or pancreatic origin [13].

Schwannomas and solitary neurofibromas are often indistinguishable on CSIs. Typically, RBNSTs manifest as round or oval lesions, different from BNSTs located in the extremities that usually acquire a spindle shape; this disparity arises since RBNSTs affect smaller peripheral nerves or nerve plexuses while those located in the extremities are contiguous with a specific nerve [13].

In general, on CT they appear as hypodense lesions (20–40 Hounsfield Units), minimally enhancing in the dynamic phases [1]. MRI characteristics are also non-specific: intermediate signal on T1-weighted images, hyperintensity on T2-weighted images, and variable contrast enhancement [3, 14].

**Fig. 1:**
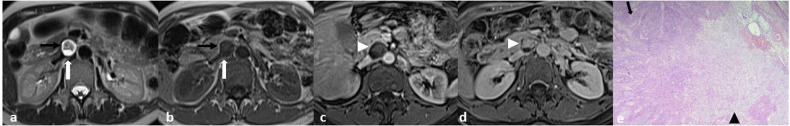
**a**–**d** MR images in a 46-year-old woman with a peripancreatic round mass determining slight compression of the inferior vena cava. The lesion has a heterogenous signal on both T2-weighted (**a**) and T1-weighted (**b**) images due to the presence of cystic (white arrows) and solid (black arrows) components. The solid component has progressive contrast enhancement (white arrowheads in **c** and **d**). These findings were suspicious for mucinous cystadenomas of the pancreatic head. **e** Final histology reveals intermixed “Antoni A” (on the left, black arrow) and “Antoni B” (on the right, black arrowhead) areas (Hematoxylin-eosin stain; original magnification, × 40), compatible with Schwannoma

**Fig. 2:**
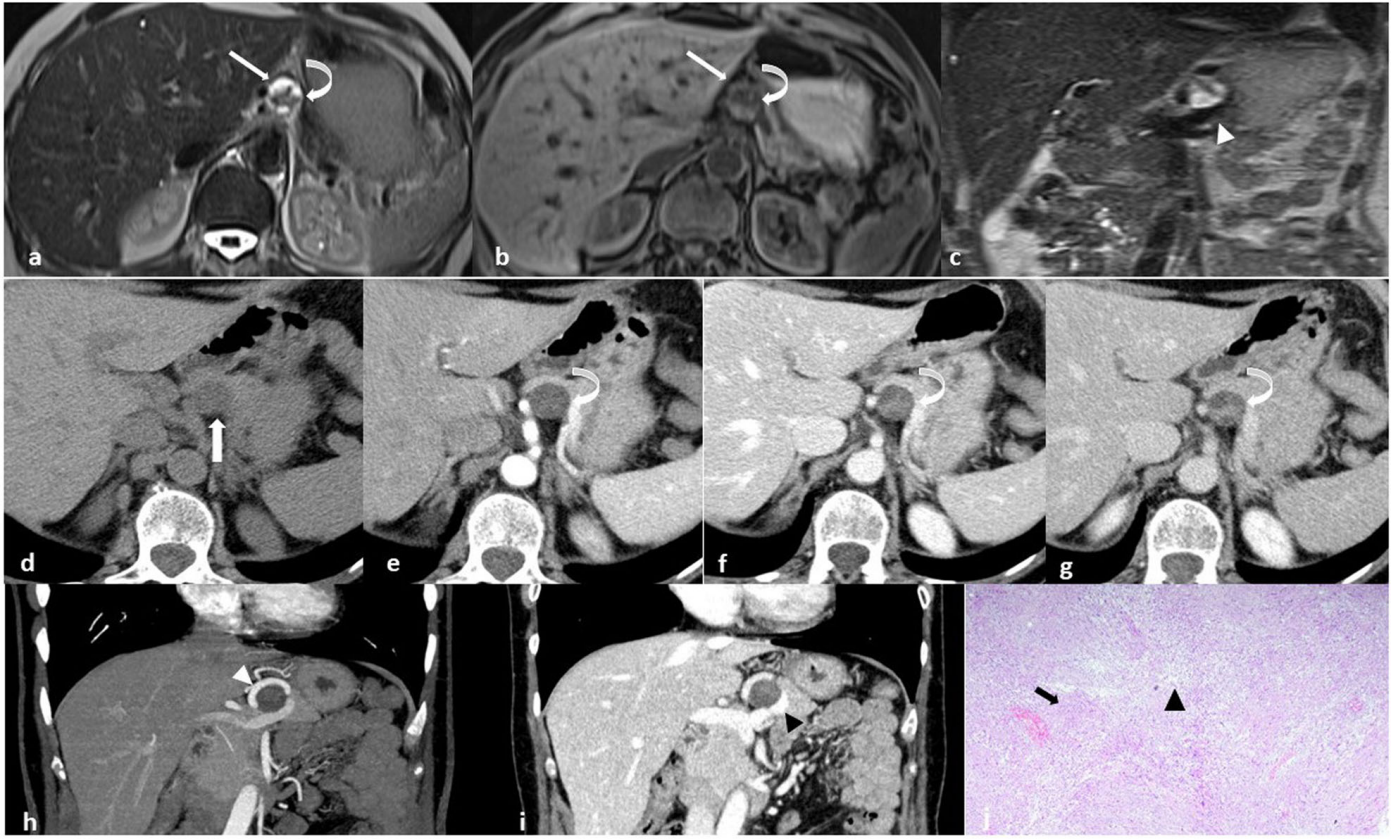
**a**, **b** Axial MR images of a patient with incidentally discovered small peripancreatic round mass: T2-weighted (**a**) and T1-weighted fat-sat (**b**) sequences show mixed intralesional solid (curved arrows) and cystic (straight arrows) components. **c** Coronal T2w image demonstrates the close relationship with the splenic vein (white arrowhead). **d**, **e** Axial CT images of the same patient: before contrast medium administration the lesion (arrow) is hypo-isodense (**d**); contrast enhancement appears progressive and inhomogeneous providing the lesion a pseudocystic appearance due to the presence of a solid inner component (curved arrow, **e**–**g**). **h**, **i** Coronal maximum intensity projection (MIP) (**h**) and multiplanar reconstructed (MPR) (**i**) CT images better show the close relationship with the splenic artery (white arrowhead, (**h**) and vein (black arrowhead, **i**), both encircling the lesion. **j** At microscopic examination, the mass exhibits an encapsulated proliferation of spindle cells in a storiform pattern (black arrow) with focal nuclear palisades (Verocay bodies in an Antoni A area) and areas of cystic degeneration (Antoni B, black arrowhead), compatible with Schwannoma (Hematoxylin-eosin stain; original magnification, × 40); S-100 was strongly and diffusely expressed by tumor cells (not shown)

**Fig. 3:**
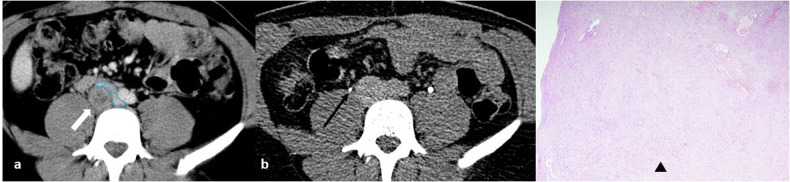
**a** Axial contrast-enhanced CT image in a 28-year-old man shows a retrocaval, round, solid mass (white arrow) with heterogeneous enhancement due to the presence of intralesional necrosis; the mass compresses the inferior vena cava (blue line) without evidence of thrombosis. **b** Axial delayed-phase CT image shows ureteral opacification, with the right ureter running close to the mass without encasement or obstruction (black arrow). **c** On microscopic examination, proliferating spindle tumor cells arranged in a fascicular fashion and accompanied by collagenous fibers (black arrowhead) are observed (Hematoxylin-eosin stain; original magnification, × 40); immunohistochemically, the tumor cells were strongly positive for S-100 protein and the Ki67-index was about 3% (not shown)

**Fig. 4:**
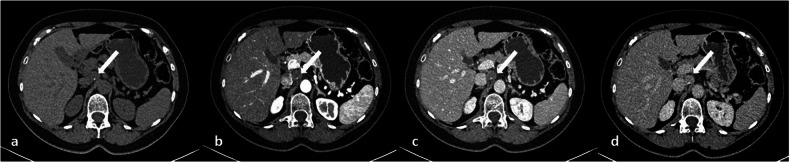
**a**–**d** CT images in a 64-year-old woman with a history of breast cancer incidentally reveal a round, paracaval solitary mass (white arrows); in the non-contrast-enhanced image (**a**) the mass appears solid and isodense with small, focal, inner calcification; after contrast medium administration, progressive enhancement is visible (**b**–**d**)

**Fig. 5:**
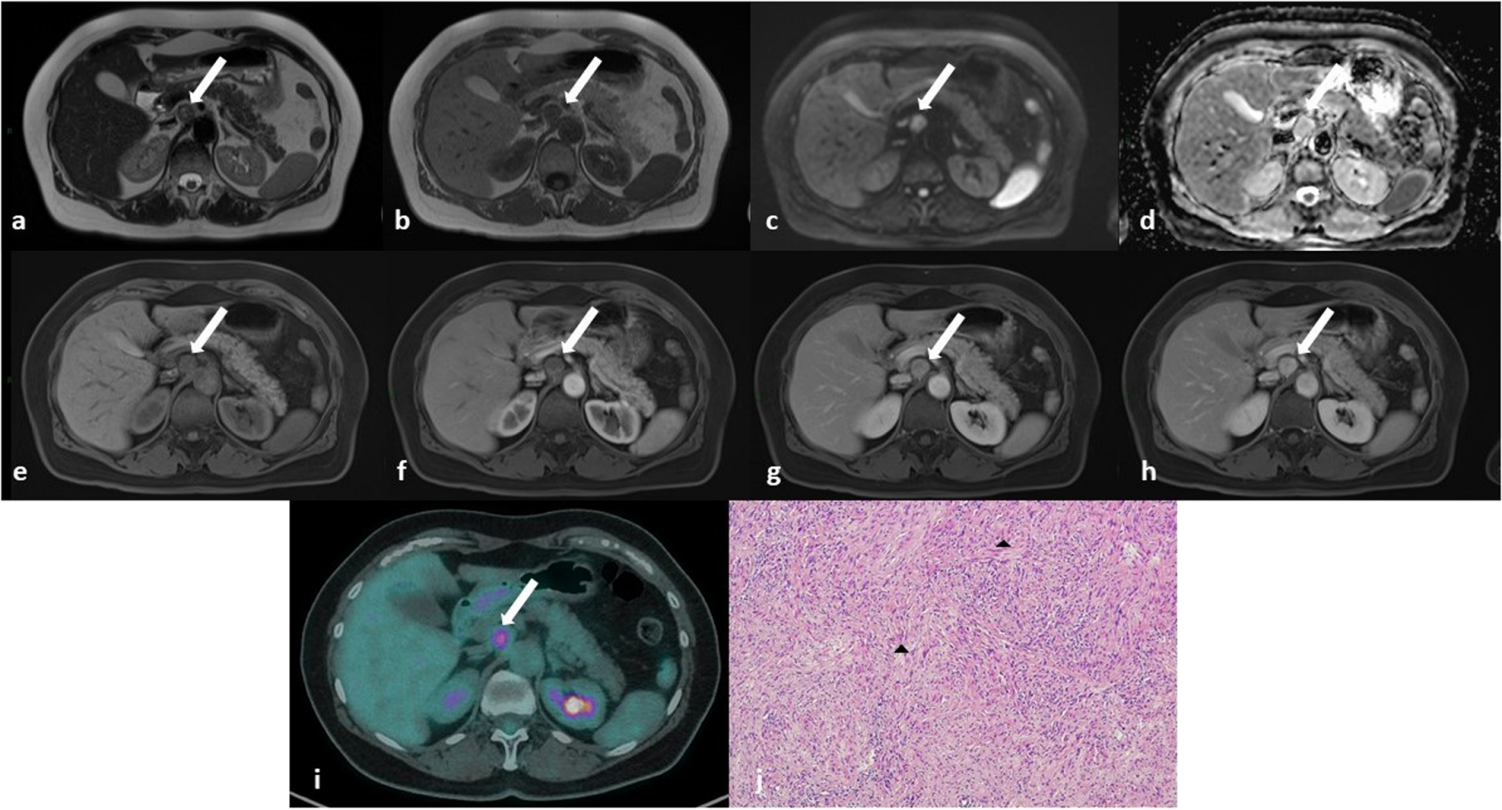
Same patient as in Fig. 4. **a**–**h**) MR images show a round mass (white arrows) with slightly hyperintense signal on T2-weighted images due to focal cystic degeneration (**a**), intermediate signal on T1-weighted images (**b**), high signal on diffusion-weighted images with the highest *b* value (**c**) associated with elevated apparent diffusion coefficient (ADC) signal (**d**). The mass is characterized by progressive contrast enhancement (**f**–**h**). **i** On 18F-FDG PET/CT, the lesion shows a central area of hypercaptation (white arrow). **j** After surgical removal, the final histology was a hypercellular Schwannoma (Antoni A): tumor cells are spindle and wavy (black arrowheads) with tapered ends and show ill-defined cytoplasm (Hematoxylin-eosin stain; original magnification, × 40)

On imaging, Schwannomas may show two distinct components that reflect their histology: the myxoid-rich Antoni B areas appear hypodense on CT, hyperintense on T2-weighted, and hypointense on T1-weighted MRI, with poor contrast enhancement; the Antoni A areas with compact cells display relatively high density on CT, hypointense signal on T2-weighted MRI, and gradually progressive enhancement. These features result in a heterogeneous pattern on post-contrast CT or MRI (Figs. 1–5) [15].

**Fig. 6:**
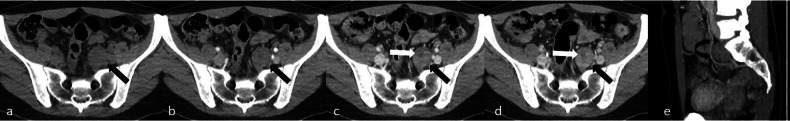
**a**–**d** Axial non-contrast (**a**) and contrast-enhanced (**b**, **c**, **d**) CT images show a left pre-sacral, oval mass (black arrows), with progressive and heterogeneous contrast enhancement due to intralesional necrosis (white arrows in **c** and **d**). Sagittal MIP reconstruction (**e**) shows the left ovarian artery (black curved arrow) displaced by the mass

**Fig. 7:**
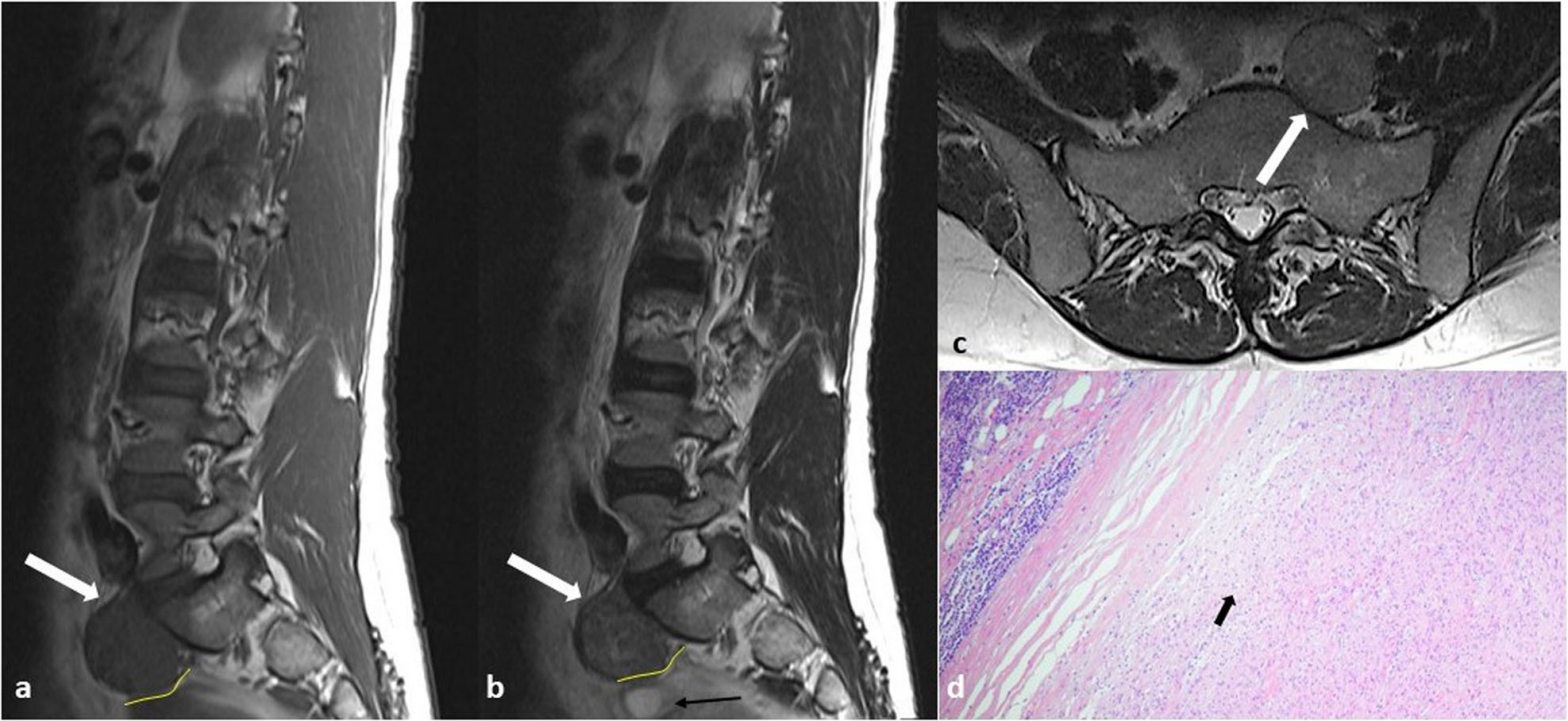
Same patient as in Fig. 6. **a**–**c** Sagittal T1-weighted (**a**) and T2-weighted (**b**) MR images and axial T2-weighted (**c**) image show a well-defined pre-sacral solid mass (white arrows), encased by a thin fibrous pseudocapsule; fatty cleavage (yellow line in **a** and **b**) separates the mass from the left ovary (black arrow in **b**). **d** Low-power photomicrograph shows representative histological features of neurofibroma: a hypocellular proliferation (black arrow) composed of interlacing bundles of elongated bland cells with wavy nuclei and interspersed collagen fibrils. No Verocay bodies or nuclear palisading are seen (Hematoxylin-eosin stain; original magnification, × 40)

**Fig. 8:**
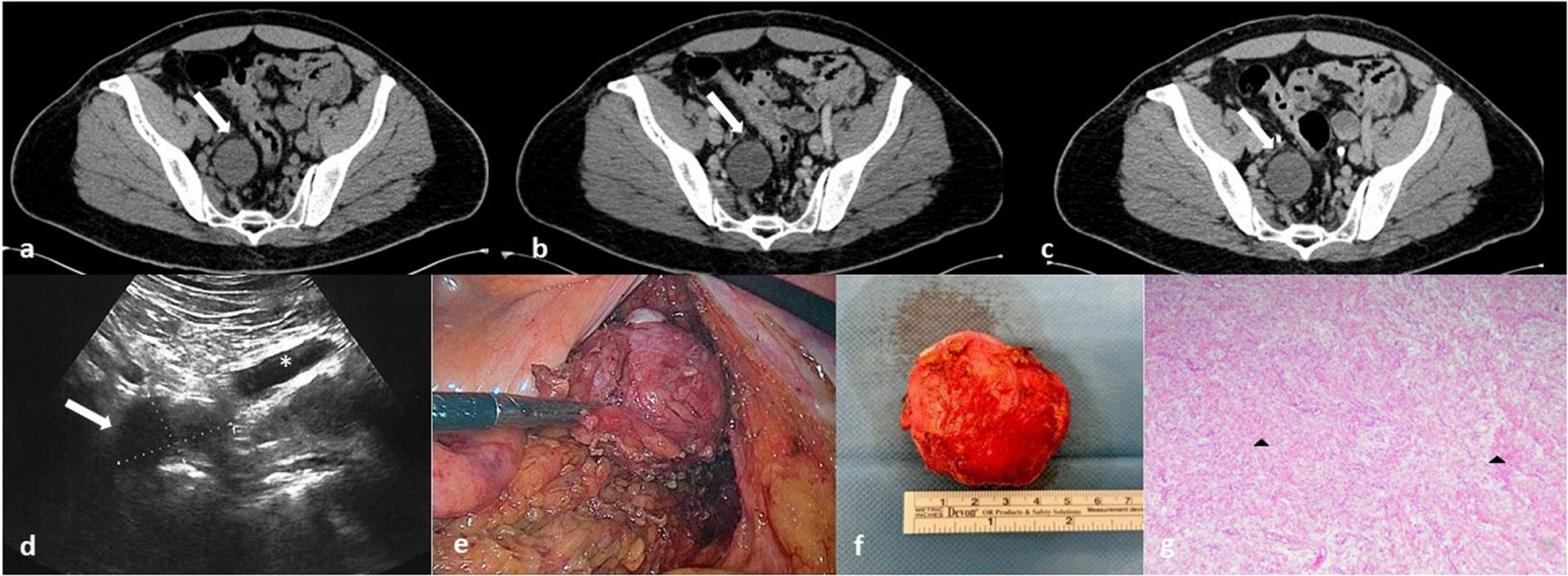
**a**–**c** Axial CT images (**a** non-contrast, **b** and **c** contrast-enhanced) show a well-defined, oval, right-sided pelvic mass (white arrows); this hypodense mass appears well circumscribed, with slight peripheral contrast enhancement on the ventral portion; no compression on surrounding structures is visible. **d** Ultrasound shows a well-defined, round, homogeneously hypoechoic pelvic mass (white arrow) adjacent to the right external iliac artery (white asterisk) (courtesy of Dr. Ravetta). **e** Intraoperative image of laparoscopic excision of the mass. **f** Photograph of gross pathologic specimen confirms that the mass seen in (**a**–**c**) is well-circumscribed with a solid and homogeneous cut surface. **g** Low-power photomicrograph demonstrates a proliferation of spindle cells with tapered and wavy nuclei (black arrowheads), indistinct cytoplasmic borders arranged in fascicles (Hematoxylin-eosin stain; original magnification, × 40), compatible with neurofibroma

Similarly, the enhancement pattern in neurofibromas reflects their underlying histological features. Tumors characterized pathologically by a hypocellular proliferation of interlacing bundles of elongated bland cells often display heterogeneous contrast enhancement (Figs. 6–7). Conversely, tumors characterized by a highly cellular proliferation of spindle cells typically exhibit homogeneous contrast enhancement (Fig. 8) [1].

**Fig. 9:**
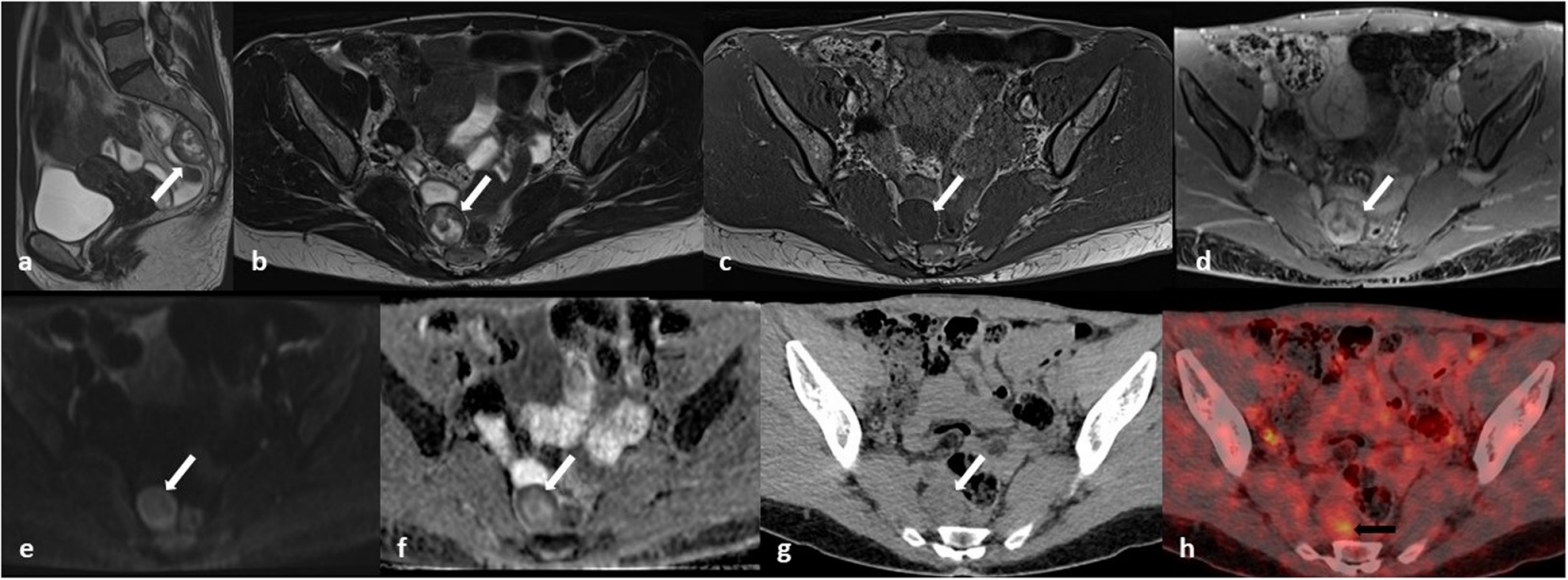
Retroperitoneal Schwannoma in a 52-year-old woman. **a**–**f** MR images show a round encapsulated mass (white arrows) with a central area of hypointense signal and peripheral hyperintensity on T2-weighted images (target sign) (**a**–**b**), intermediate signal on T1-weighted images (**c**), inhomogeneous contrast enhancement on fat-suppressed gadolinium-enhanced T1-weighted images, with a central focal area of contrast enhancement surrounded by peripheral hypointensity and an outer area of enhancement (**d**); moderate signal on diffusion-weighted images with the highest *b* value (**e**) associated with a peripheral hypointensity on apparent diffusion coefficient (ADC) map (**f**). **g** On non-enhanced CT, the lesion appears homogeneously isodense to the muscles. **h** On 18F-FDG PET/CT, it shows a focal area of hypercaptation (black arrow)

Neurofibromas and, less frequently, Schwannomas may show the “target sign”, characterized by a central area of hyperdensity surrounded by a peripheral hypodensity on CT scans, while on MRI it consists of a central area of hypointensity and peripheral hyperintensity on T2-weighted images or a central focal area of contrast enhancement and peripheral hypointensity on gadolinium-enhanced T1-weighted images (Fig. 9) [16].

RBNSTs may also demonstrate the “fascicular sign”, i.e., numerous small ring-like structures with peripheral hyperintensity at T2-weighted MRI, that most likely represent the fascicular bundles within the nerves, however, this sign has been described also in the case of well-differentiated malignant nerve sheath tumors [1, 17].

**Fig. 10:**
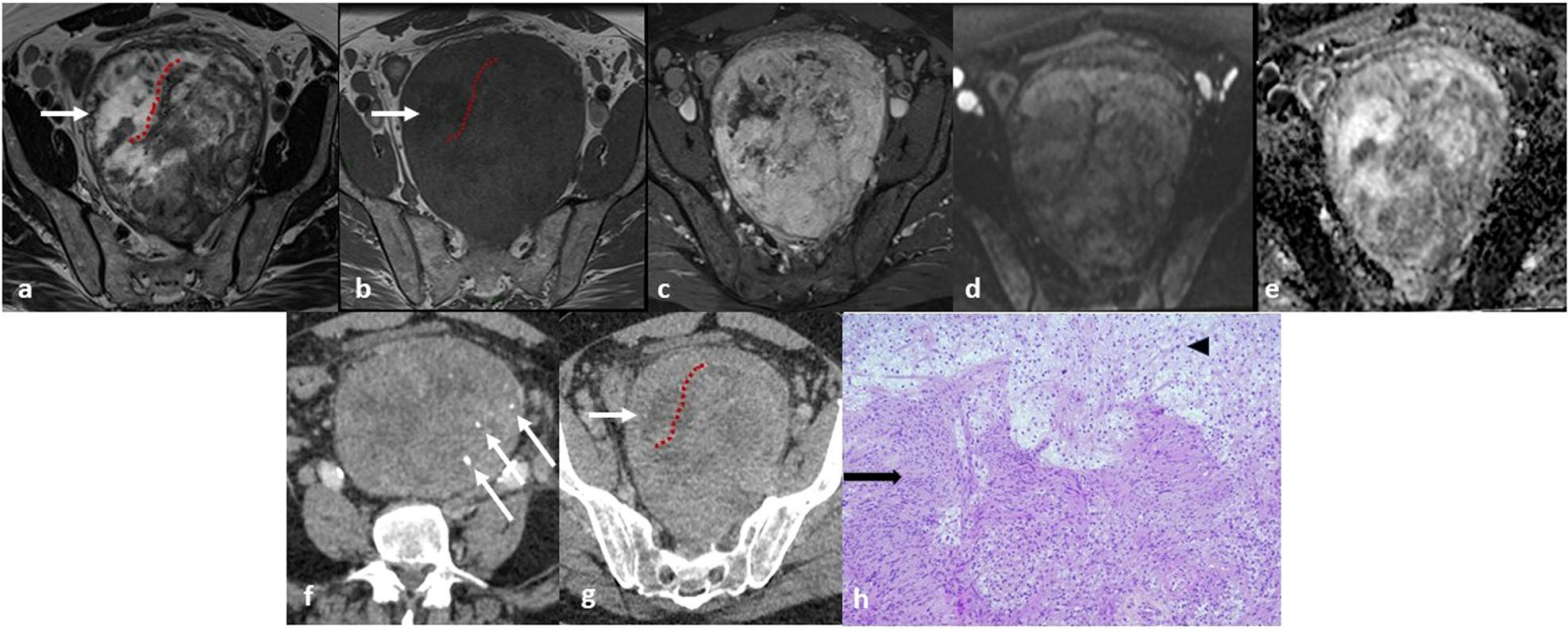
**a**–**e** Magnetic resonance (MR) images of a 63-year-old man with a voluminous pre-sacral mass: on T2-weighted (**a**) and T1-weighted (**b**) images the lesion appears grossly inhomogeneous with signs of internal cystic degeneration (white arrows) and septa (red dotted lines); heterogenous enhancement is seen after contrast medium administration (**c**); no hyperintense signal is seen in diffusion-weighted imaging (DWI) (**d**) nor pathological signal restriction on apparent diffusion coefficient (ADC) sequence (**e**). **f**, **g** Contrast-enhanced computed tomography (CT) images of the same lesion showing rare, focal inner calcifications (**f**, white arrows) and similar features of cystic degeneration and septa seen on MRI (**g**). The mass has clear, regular boundaries and no infiltration of the surrounding structures is detectable at any level suggesting its expansive behavior. **h** Histology reveals the proliferation of predominantly spindle cells, with no significant cytologic atypia or increased mitotic activity. Hypercellular (Antoni A, lower part, black arrow) and hypocellular (Antoni B, upper part, black arrowhead) areas are seen. Immunohistochemistry revealed diffuse positivity for S-100 and a low Ki67 proliferation index (not shown) consistent with Schwannoma

In addition, RBNSTs’ density and intensity depend on spontaneous intralesional rearrangements [18, 19], such as cystic degeneration, hemorrhage, necrosis, and calcifications [16, 17] (Figs. 1, 2, 4, 5, 10). While cystic degeneration is present in a significant proportion of Schwannomas, it cannot be considered a distinctive hallmark due to its occurrence in retroperitoneal sarcomas as well [17, 20].

The cystic areas appear hypodense on unenhanced CT images, hyperintense on T2-weighted MR images, and without enhancement after contrast administration. Hemorrhagic foci should be suspected based on intralesional hyperintense areas on T1-weighted MR images. Calcifications may be punctate, mottled, or curvilinear and, when present, are usually localized on the peripheral region of the tumor [3].

When performed, 18F-fluorodeoxyglucose-positron emission tomography/CT (FDG-PET/CT) reveals FDG accumulation in the solid components of RBNSTs (Figs. 5, 9).

## Differential diagnosis on cross-sectional investigations

The differential diagnosis between RBNSTs and other retroperitoneal neoplasms on CSIs is challenging, and sometimes a precise differentiation is not possible. A comprehensive analysis of the mass’s location, origin, imaging features, and associated clinical factors can be of help. Nevertheless, histological examination serves as the definitive method to confirm the tumor’s nature, ultimately guiding the treatment strategy.

The main conditions to consider during the diagnostic evaluation of a retroperitoneal mass include carcinoma metastasis, adenopathy, lymphoid neoplasms (such as lymphomas and Castleman disease), extragonadal germ cell tumors (including seminoma and non-seminomatous tumors like embryonal carcinoma, choriocarcinoma, and teratomas), malignant peripheral nerve sheath tumors, neurogenic tumors of non-neural sheath origin (such as paragangliomas, ganglioneuroma, and ganglioneuroblastoma), vestigial cystic tumors, and other mesenchymal tumors (such as adipose tissue tumors, smooth muscle tumors, fibroblastic and myofibroblastic tumors, striated muscle tumors, vascular tumors, extraskeletal osseous and cartilaginous tumors, tumors with uncertain differentiation, and unclassified/undifferentiated sarcomas). Table 1 outlines the main categories of retroperitoneal masses in adults.

**Table 1:** Main categories of retroperitoneal masses in adults

Categories of retroperitoneal masses in adults
**Solid – Neoplastic**
Liposarcoma
Leiomyosarcoma
Lymphoma
Neurogenic tumors
Germ-cell tumors
**Solid – Non-neoplastic**
Retroperitoneal fibrosis
Extramedullary hematopoiesis
Erdheim-Chester disease
**Cystic – Neoplastic**
Cystadenoma
Lymphangioma
Cystadenocarcinoma
Teratoma
**Cystic – Non-neoplastic**
Mullerian cyst
Epidermoid cyst
Lymphocele
Hematoma

First, the location and origin of the mass is crucial. Schwannomas and neurofibromas typically arise from peripheral nerves and are often seen along the course of these nerves. They tend to displace rather than invade adjacent structures (Figs. 3, 6), in contrast, other retroperitoneal neoplasms, such as adrenal tumors, renal cell carcinomas, or lymphomas, tend to infiltrate neighboring structures or have distinct organ-based origins. Moreover, RBNSTs tend to grow slowly over time, whereas malignant retroperitoneal neoplasms typically exhibit more rapid growth.

Second, the appearance of the lesion on imaging is important. As previously stated, RBNSTs often present as well-circumscribed, encapsulated masses with a variable degree of enhancement on contrast-enhanced MRI and CT scans. Other retroperitoneal neoplasms exhibit different imaging characteristics depending on their tissue of origin; for example, adrenal tumors may have a lipid component, while renal cell carcinomas usually are hypervascular. Retroperitoneal malignancies like sarcomas or lymphomas may exhibit areas of necrosis, hemorrhage, or calcifications, however, these findings are not specific (Figs. S1–S3).

The avid uptake of FDG typically shown by these tumors, which is in contrast with their harmless nature, should be kept in mind in the differential diagnosis of a retroperitoneal mass incidentally detected on PET. However, no correlation exists between the degree of FDG avidity (low or high SUV) and potential malignancy that actually makes PET useful in differentiating RBNSTs from aggressive retroperitoneal neoplasms [21].

Lastly, ancillary findings such as the presence of NF1 in a patient can be indicative of neurofibromas, as they are often associated with this genetic condition.

## Surgical treatment

All retroperitoneal masses, including those suspected of RBNST, should be evaluated for surgical removal which is always recommended as the primary indication to correctly ascertain their nature. In most cases, diagnostic uncertainty prompts the surgeon to obtain complete radicality following the common criteria of oncological resection. Instead, when the radiological features and the clinical history are strongly suggestive of RBNST, function-sparing surgery should be pursued by enucleating the tumor from the intact nerve fascicles [22].

These factors may condition the surgical approach in terms of technique (open or minimally invasive) and anatomical route (intra or retroperitoneal). Mass-related factors such as size, position, relationships with nearby structures, and the expertise of the surgical team with the available techniques and approaches should guide decision-making at the time of surgical planning. Close proximity or direct contact with organs, viscera, or vessels should anticipate the possibility that these structures might be partially or fully involved and resected en-bloc with the mass; for this reason, patients must be accurately informed beforehand [22].

The minimally invasive approach to RBNST excision has been proven safe and feasible in expert hands with obvious advantages for patients [23]. Ideal cases are those with small to medium size masses (roughly less than 8–10 cm) and more favorable locations. However, several published reports attest that RBNSTs can be safely and effectively treated by laparoscopy even in challenging anatomical situations, such as along the course of retroperitoneal major vessels and their visceral branches [23, 24].

Laparoscopy is preferred in most cases due to its wider availability and lower costs as compared to robotic technology. The availability of robotic surgical systems may be beneficial for deeply located lesions, especially in narrow anatomical spaces where maneuverability of wristed instruments, tridimensional vision, and physiologic tremor filtering can substantially increase the ease, effectiveness, and safety of the procedure [25].

## Conclusions

In conclusion, RBNSTs can show significant histological and radiological heterogeneity. The lack of specific appearance at CSI, as well as their variable location within the retroperitoneal space, can pose challenges in distinguishing them from malignant tumors.

When solitary retroperitoneal masses are incidentally encountered, radiologists should prioritize the exclusion of potential malignancies and then they should consider RBNSTs in the differential diagnosis. While radiological features might suggest a benign neurogenic origin, definitive confirmation relies solely on histological analysis.

### Supplementary information

ELECTRONIC SUPPLEMENTARY MATERIAL

## Abbreviations

CSI: Cross-sectional imaging
CT: Computed tomography
FDG-PET: Fluorodeoxyglucose-positron emission tomography
MRI: Magnetic resonance imaging
NF1: Neurofibromatosis type 1
RBNST: Retroperitoneal benign nerve sheath tumor
US: Ultrasound
