# Supplementary material for: Correlation between imaging and histology in benign solitary retroperitoneal nerve sheath tumors: a pictorial review
Source: Insights Imaging. 2024 May 31;15:132. doi: 10.1186/s13244-024-01709-5 (PMC11139841; doi:10.1186/s13244-024-01709-5)
Supplement: Supplementary file 1 — ELECTRONIC SUPPLEMENTARY MATERIAL [file 13244_2024_1709_MOESM1_ESM.pdf]

Correlation between imaging and histology in benign solitary retroperitoneal nerve sheath tumors: a pictorial review

ELECTRONIC SUPPLEMENTARY MATERIAL

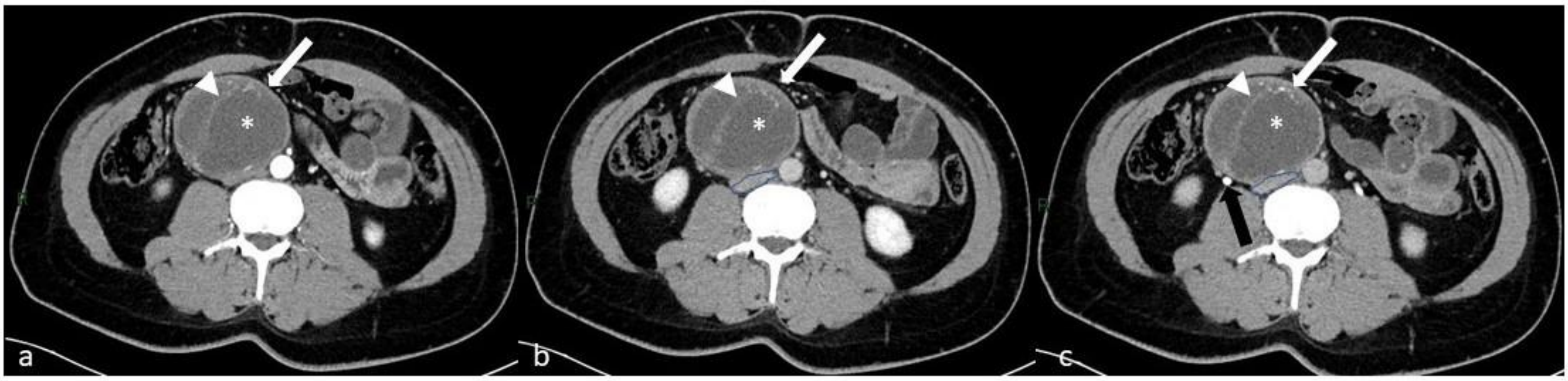

Figure S1. Extragonadal germ cell tumor (seminoma) in a 40-year-old man. a-c) Axial contrast-enhanced CT images show a large precaval oval mass with cystic components (white asterisks), surrounded by enhancing irregular solid tissue (white arrows) and intralesional thick septa (white arrowheads). The lesion compresses the inferior vena cava (blue line in b and c) and displaces the right ureter (black arrow in c).

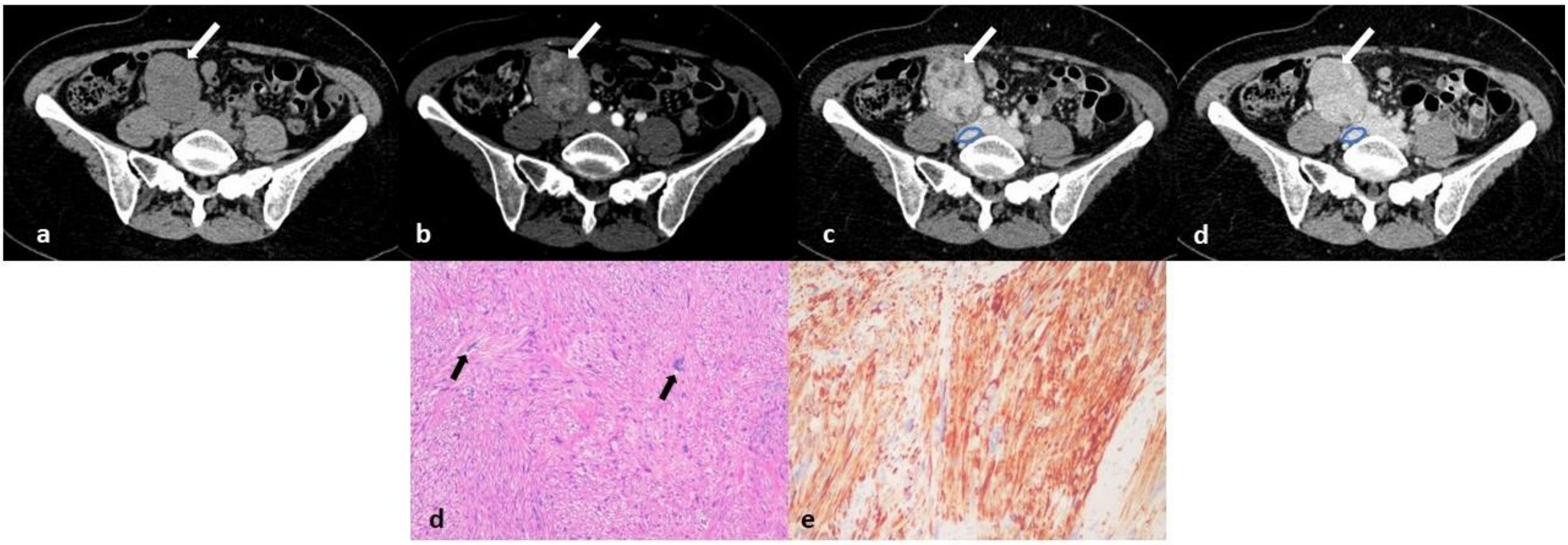

Figure S2. Retroperitoneal leiomyosarcoma in a 59-year-old woman. a-d) Axial contrast-enhanced CT images show a large precaval solid oval mass (white arrows) characterized by intense, inhomogeneous, progressive contrast enhancement (b-d). The lesion slightly compresses the inferior vena cava (blue line in c and d). d-e) Leiomyosarcoma is characterized by spindle-shaped cells with atypical, blunt-ended nuclei (black arrows) and eosinophilic fibrillary cytoplasm, arranged in intersecting fascicles. Tumor cells express smooth muscle markers, such as desmin.

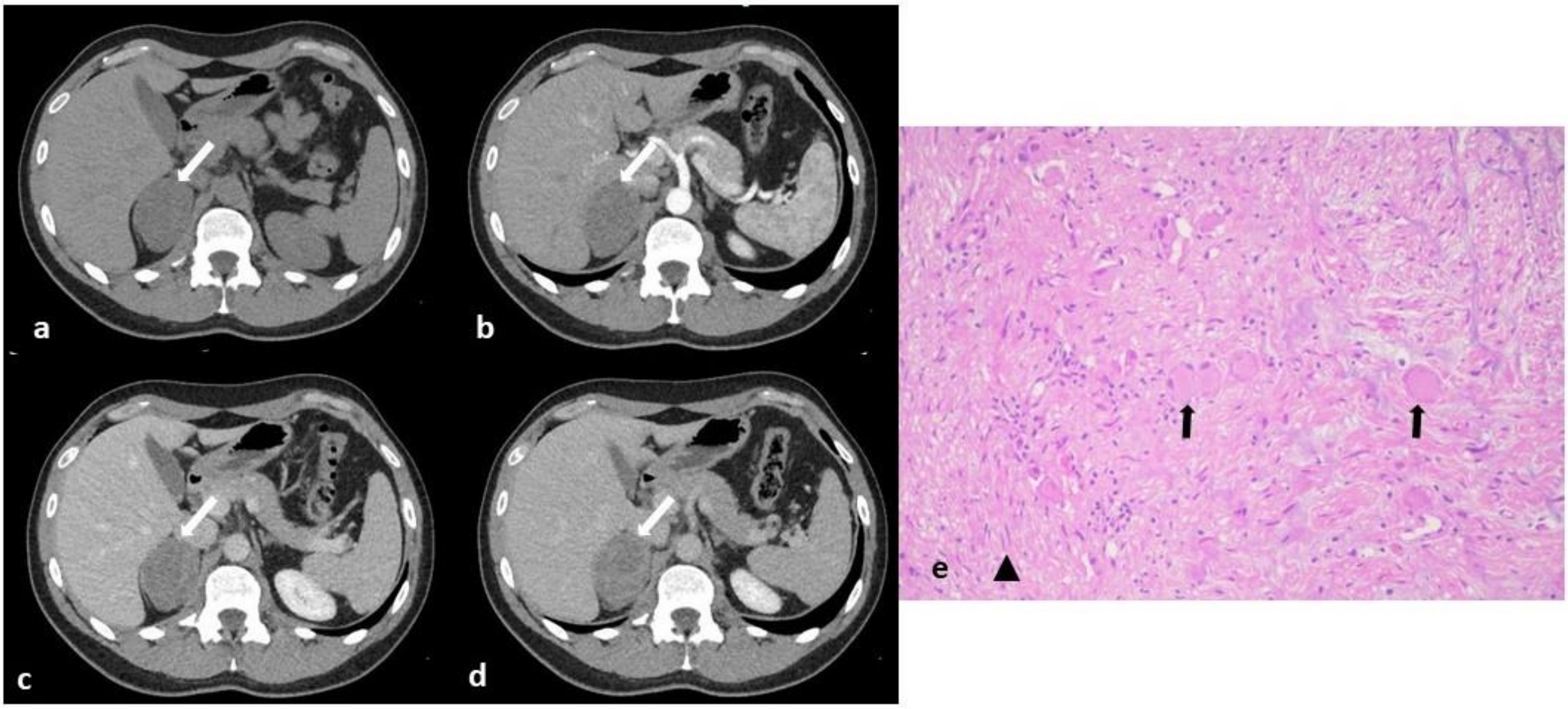

Figure S3. Retroperitoneal ganglioneuroma in a 33-year-old man. a-d) Axial contrast-enhanced CT images show a large solid oval mass in the right adrenal lodge (white arrows) characterized by mild contrast enhancement in the late phases (c-d). e) Ganglioneuroma is characterized by an admixture of scattered ganglion cells (round-to-polygonal large cells with eosinophilic cytoplasm, black arrows) and spindle Schwann cells (black arrowhead).
